# LAI estimation through remotely sensed NDVI following hail defoliation in maize (*Zea mays* L.) using Sentinel-2 and UAV imagery

**DOI:** 10.1007/s11119-023-09993-9

**Published:** 2023-02-27

**Authors:** Jacopo Furlanetto, Nicola Dal Ferro, Matteo Longo, Luigi Sartori, Riccardo Polese, Daniele Caceffo, Lorenzo Nicoli, Francesco Morari

**Affiliations:** 1 TESAF Department, University of Padova, Legnaro, 35020 Padua, Italy; 2 DAFNAE Department, University of Padova, Legnaro, 35020 Padua, Italy; 3 Società Cattolica di Assicurazione S.p.A., 37126 Verona, Italy

**Keywords:** LAI, UAV, Hail, Remote sensing, Insurance

## Abstract

Extreme events such as hailstorms are a cause for concern in agriculture, leading to both economic and food supply losses. Traditional damage estimation techniques have recently been called into question since damages have rarely been quantified at the large-field scale. Damage-estimation methods used by field inspectors are complex and sometimes subjective and hardly account for damage spatial variability. In this work, a normalized difference vegetation index (NDVI)-based parametric method was applied using both unmanned aerial vehicles (UAV) and Sentinel-2 sensors to estimate the leaf area index (LAI) of maize (*Zea mays* L.) resulting from simulated hail damage. These methods were then compared to the LAI values generated from the Sentinel-2 Biophysical Processor. A two-year experiment (2020–2021) was conducted during the maize cropping season, with hail events simulated during a range of maize developmental stages (the 8th-leaf, flowering, milky and dough stages) using a 0–40% defoliation gradient of damage intensities performed with the aid of specifically designed prototype machines. The results showed that both sensors were able to accurately estimate LAI in a nonstandard damaged canopy while requiring only the calibration of the extinction coefficient $k(\vartheta )$ in the case of parametric estimations. In this case, the calibration was performed using 2020 data, providing $k(\vartheta )$ values of 0.59 for Sentinel-2 and 0.37 for the UAV sensor. The validation was performed on 2021 data, and showed that the UAV sensor had the best accuracy (R^2^ of 0.86, root-mean-square error (RMSE) of 0.71). The $k(\vartheta )$ value proved to be sensor-specific, accounting for the NDVI retrieval differences likely caused by the different spatial operational scales of the two sensors. NDVI proved effective in parametrically estimating maize LAI under damaged canopy conditions at different defoliation degrees. The parametric method matched the Sentinel-2 biophysical process-generated LAI well, leading to less underestimations and more accurate LAI retrieval.

**Supplementary Information:**

The online version contains supplementary material available at 10.1007/s11119-023-09993-9.

## Introduction

Extreme weather events are globally responsible for considerable economic losses in the agricultural sector, quantified at $20 billion in 2007 (Mahul & Stutley, 2010). In particular, considerable crop-yield and forage-quality losses are caused by hailstorms (de Leeuw et al., 2014; Gobbo et al., 2021; Roth & Lauer, 2008; Shekoofa et al., 2012; Zhao et al., 2012), the frequency and intensity of which are expected to increase in Europe, driven by climate change (Hov et al., 2013; Munich RE, 2017).

Insurance companies have a long history of assessing hail damages to crops, but some known limitations exist (de Leeuw et al., 2014; Peters et al., 2000): (1) assessments are conducted mainly by field surveyors in small areas compared to the entire hailstorm swaths, which can have lengths from 80 to 330 km and widths from 10 to 25 km (Bell et al., 2020); (2) hailstorm intensities exhibit strong spatial variability (Nisi et al., 2016), making accurate damage estimations difficult (Bell & Molthan, 2016); (3) inspections are time-consuming, queueing up when simultaneous hailstorms occur and increasing the risk of compromising precise assessments (Furlanetto et al., 2021); (4) evaluations of hail damage have a certain degree of subjectivity (Zhou et al., 2016).

Satellites can improve hail damage quantification methods by providing wide-area coverage, consistent revisit times and objective measurements. In recent years, unmanned aerial vehicles (UAVs) have also proven to be effective in assessing damages due to their relatively high ground resolutions (Zhang et al., 2021), although UAV data suffer from limited area coverage, potential uncertainties, e.g., due to the illumination conditions during flight (Abdelbaki et al., 2021), and demanding planning.

Estimating the leaf area index (LAI) using remote sensing is pivotal for accounting for hailstorm-related effects, such as crop defoliation (Shekoofa et al., 2012; Vescovo et al., 2016), growth, and, finally, yield. In fact, in many crops such as maize (*Zea mays* L.), hail damage evaluations have historically relied mostly on the defoliation degrees at different phenological stages (Shapiro et al., 1986; Zhao et al., 2012), in addition to damages to reproductive organs, ears, or direct grain losses (Gobbo et al., 2021). Nonetheless, the relationship between the LAI and yield loss is not trivial. As pointed out by Lauer et al., (2004), there is no linear relationship between defoliation and yield reduction in maize, even when different defoliation levels are applied at the same plant stage. Moreover, different plant stages are differently affected by defoliation, with higher losses during the reproductive phases (USDA, 2020). Shekoofa et al. (2012) confirmed that defoliation led to greater yield losses when it occurred 25 days after the silking stage compared to 35 days after this stage. Different remote sensing LAI estimation techniques have been developed in recent years, relying on a wide array of sensors, namely, light detection and ranging (LiDAR), synthetic aperture radar (SAR) and optical sensors. The LiDAR technique is based on a laser pulse and on the analysis of the backscattered signal. LiDAR data have eventually allowed researchers to define object distances at resolutions within a few centimetres, thus providing canopy height information, 3D models and signal-return patterns in accordance with different defoliation levels. LAI estimations using LiDAR data have been explored in a few studies, e.g., in maize by Vescovo et al. (2016), and LiDAR data allowed for the retrieval of the defoliation degree over hail-damaged fields. This technique, though promising, also poses a few challenges, such as the relatively small area coverage, saturation problems and the need for relatively high-density points in dense crop canopies (Li et al., 2015). SAR techniques have been explored mostly with the Sentinel-1 satellite platform. SAR data from Sentinel-1 have been used to map hailstorm swaths over croplands and have been coupled with normalized difference vegetation index (NDVI) data using backscatter analysis (Bell et al., 2019, 2020). In these two studies, this technique appeared to be suitable and capable of identifying damaged areas by analysing sudden changes in the backscattered signal following defoliation. This approach is still new and poses some challenges mainly related to, e.g., the interpretation of stand-alone signal responses. While the NDVI seems to follow a coherent decrease following damages, SAR does not appear to exhibit a similar pattern. Moreover, SAR signal is strongly influenced by wet ground conditions and can become saturated when crops are fully developed (Bériaux et al., 2015). Four main classes of retrieval techniques from optical remotely sensed images have been used for LAI estimations over the years (Verrelst et al., 2019). Parametric regression methods rely on the explicit relationships between LAI and vegetation indices (VIs) (Ali et al., 2015; Brogi et al., 2020; Kaplan & Rozenstein, 2021; Xing et al., 2020). Linear and nonlinear nonparametric regression methods, e.g., Gaussian Process Regression (Rivera-Caicedo et al., 2017), artificial neural networks (Chen et al., 2020), random forests (Abdelbaki et al., 2021), and support vector machines (Tuia et al., 2011; Zhang et al., 2021), do not assume any explicit relationship between LAI and spectral reflectance. Radiative transfer models (RTMs) rely on deterministic physical-based models to simulate radiative processes at the canopy level from which LAI can be estimated (Zhu et al., 2019). Finally, hybrid methods combine nonparametric and physical-based approaches, e.g., through inversion processes (Duan et al., 2014; Zhu et al., 2019). Compared to other techniques, parametric regression methods offer a nonsite-specific and relatively simple way to assess LAI (Ali et al., 2015; Brogi et al., 2020; Walthall et al., 2004), thus preventing the need for ground data or a priori knowledge as is required in RTMs. However, parametric regression methods still suffer from spatiotemporal variabilities in the results due to the crop status and growth stage, soil conditions (Levitan et al., 2019) and reflectance measurement uncertainties (Walthall et al., 2004). While parametric regression methods can benefit from hyperspectral sensors’ capacities to exploit the full spectrum (Tanaka et al., 2015; Xing et al., 2020), their relatively simple formulations best correspond to the abilities of multispectral sensors onboard either satellites or UAVs.

Among the developed parametric regression methods, those using NDVI (Tucker, 1979) have been confirmed to be straightforward and effective for remote LAI estimations in various crops, such as maize, soybean (*Glycine max* L.), potato (*Solanum tuberosum* L.) and wheat (*Triticum aestivum* L.) (Ali et al., 2015; Brogi et al., 2020; Kaplan & Rozenstein, 2021; Walthall et al., 2004). Some limitations in the use of NDVI have been reported, such as its pronounced sensitivity to the background soil conditions (Feng et al., 2019) and ineffective estimates when saturation occurs (e.g., at crop LAI values > 4) (Xing et al., 2020), although recent findings by Kaplan and Rozenstein (2021) have suggested that narrowing the near-infrared (NIR) bands during the NDVI formulation process can help in reducing the saturation effect.

Previous studies have focused on mapping hail damage using NDVI (e.g., Bell & Molthan, 2016; Prabhakar et al., 2019; Zhao et al., 2012), although its potential for estimating LAI—a proxy to quantify the crop growth status following damage—has not been fully exploited (Smith et al., 2005).

The goals of this study are threefold: (1) to validate a parametric NDVI model for predicting LAI damage at different hailstorm intensities and phenological stages and compare the results against machine learning-generated LAI from the Sentinel-2 Biophysical Processor; (2) to compare the LAI results obtained with multispectral sensors using two different supports and scales (satellite and UAV) and two different LAI estimation methods (parametric and machine learning-based); (3) to provide a more objective tool with which insurance companies can map and quantify hailstorm damages at the field scale.

## Materials and methods

### Experimental design and treatments

A two-year experiment (2020–2021) was conducted at a farm located in the Venice Lagoon watershed (NE Italy, 45°33′2″ N, 12°25′50″ E). The local climate is subhumid, with a mean annual temperature of 14 °C and a total annual rainfall of 1080 mm, uniformly distributed throughout the year. The reference evapotranspiration (ET_0_) averages 996 mm yr^−1^, exceeding the rainfall amount from April to September. The soil is an Endogleyic Calcisol (WRB, 2014), silty-clay-loam, characterized by low soil organic carbon (1.52 ± 0.20 g 100 g^−1^) and total nitrogen (0.21 ± 0.02 g 100 g^−1^) content. A 21-ha area was cultivated with maize in both years, with sowing occurring on March 14 (2020) and April 22 (2021) and harvesting performed on September 29 (2020) and September 22 (2021). Cropping operations —which were the same in both years—included mouldboard ploughing (0.30 m deep) followed by harrowing before seeding. Fertilizations consisted of 50 kg N ha^−1^ diammonium phosphate at seeding and 230 kg N ha^−1^ top-dress urea in late May/beginning of June. Pests and weeds were controlled with agrochemicals depending on established crop and seasonal trends. The site has a shallow water table that was maintained at an average depth of 0.80 m for subsurface irrigation during summer, eventually integrated with additional hose-reel sprinkler irrigation when the groundwater level was > 1 m deep. The weather was monitored with a dedicated weather station placed on-field (Delta Ohm, Padova, Italy). The station collected temperature, solar irradiance, relative humidity, wind speed and rainfall data hourly.

**Fig. 1:**
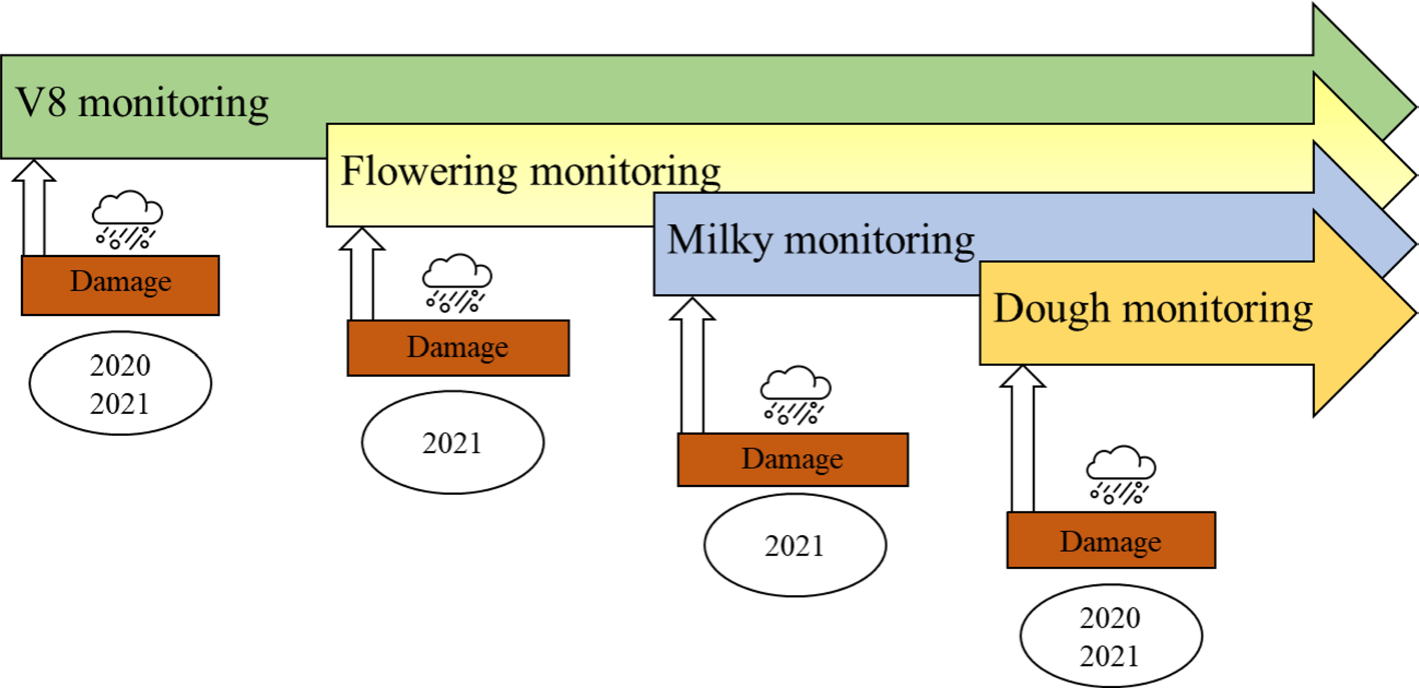
Experimental outline of damage simulations and subsequent surveys per stage adopted during both 2020 and 2021

The experimental design was conceptually the same in both 2020 and 2021 (Fig. 1). In 2020, due to the severe restrictions imposed by the COVID-19 pandemic, the experimentation was conducted on only 12 hail-damaged plots of 60 × 60 m each in two replicates plus two control plots (undamaged). The hail damages corresponding to different hail intensities (low (“Lo”), medium (“Me”), and high (“Hi”)) were tested in a factorial combination with two damage-occurrence periods, the 8th-leaf (V8) and dough (D) at stages 18 and 83–85, respectively, on the Biologische Bundesanstalt, Bundessortenamt and Chemical industry (BBCH) scale (Lorenz et al., 2001).

In 2021, each treatment was performed in triplicate in squared plots of 20 × 20 m or 60 × 60 m for a total of 36 plots plus three control plots (hereinafter CTRL) where no damage was caused (treatment-free). Four maize developmental stages were tested: 8th-leaf (V8), flowering (F), early milky (M) and dough (D) stages, corresponding to 18, 61–69, 73–79 and 83–85, respectively, on the BBCH scale (Lorenz et al., 2001). Damages were conducted from June 1–3 for the “V8” stage, from July 12–15 and July 19–20 for the “F” and “M” stages, respectively, and from August 9–10 for the “D” stage. These combinations covered a wide range of possible plant responses to damages throughout the cropping season. The different plot sizes were adopted to match the pixel ground resolution of the Landsat 8 satellite, whose images were used in the project but not reported in this study.

**Fig. 2:**
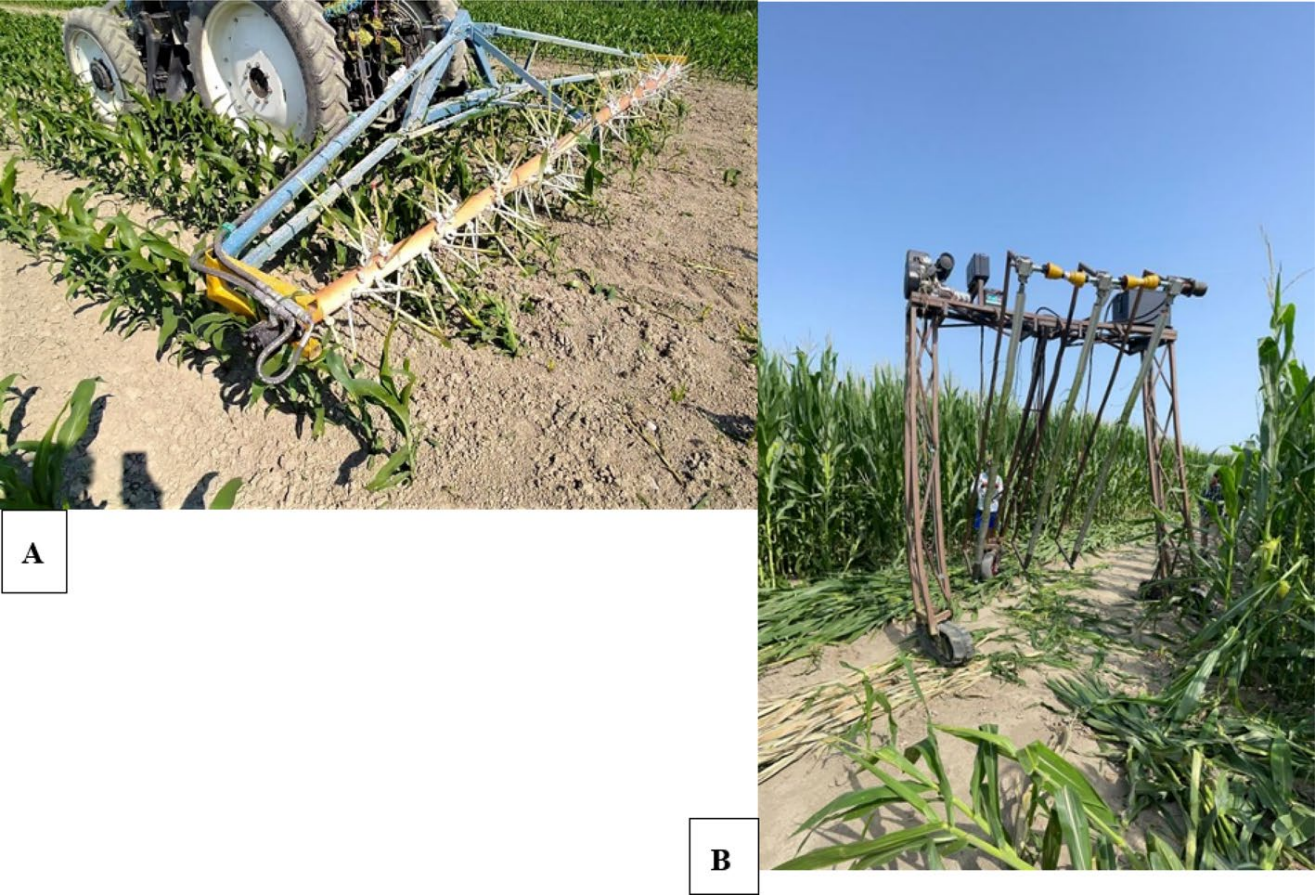
The two prototypes for simulating hail damages at different crop growth stages used during the two years of experimentation. **A** Rotating-whip prototype for damaging maize during the early growth stage (8th-leaf stage). **B** Autonomous vertical rotating pole prototype for damaging maize during the later stages (flowering, milky, and dough) *Ph. Lorenzo Carotta*

Hail damage was simulated using two prototype machines specifically designed at the University of Padova. These machines were built to match different maize heights throughout the growing phases. The first was used during the “V8” stage, consisting of a horizontal rotating pole handled by a tractor, with 0.35 m ropes attached and acting as whips; the rotating speed of the whips was regulated via an oil engine (Fig. 2A). The second machine was used for all the remaining stages. It consisted of a three-legged vertical structure equipped with vertical rotating poles attached to metal strings that whipped the maize along its height while moving forward, thanks to the dedicated engine (Fig. 2B). The crop damages were evaluated according to in-field defoliation estimates performed by field inspectors, a practice commonly adopted by insurance companies when assessing hail-caused leaf damages (Klein & Shapiro, 2011). The shape and intensity of the leaf damages caused by the prototypes effectively mimicked natural hail damages. Thanks to this, it was possible to differentiate between the “Lo”, “Me”, and “Hi” treatments within a 10–40% defoliation range.

### Soil variability characterization

The apparent electrical conductivity (EC_a_) was measured in 2020 to map the soil variability across the experimental field. A CMD Mini-Explorer (GF Instruments, Brno, Czech Republic) was used for the purpose, measuring the EC_a_ at three different depths (0–0.25 m, 0–0.50 m and 0–0.90 m). A total of 7252 data points were collected, with a 2-m interpoint distance and 20-m distance between survey lines. A global positioning system (GPS) antenna Pathfinder ProXT (Trimble Inc., Sunnyvale, CA, USA) was used to geo-reference the EC_a_ measurements by acquiring and geotagging data every second.

### On ground LAI monitoring

Ground measurements of the leaf area index (hereinafter LAI_Cept_) were conducted after every damage-simulation process at three sampling points in each plot in 2020 and two sampling points in each plot in 2021 using an Accupar LP-80 (Decagon Devices Inc., Pullman, WA, USA). This instrument is a hand-held linear photosynthetically active radiation (PAR) ceptometer. It consists of a read-out/datalogger unit and a probe containing 80 independent sensors on an 80 cm-long rod. An external PAR sensor designed to measure the above-canopy radiation can be connected to the device. Based on the above- and below-canopy PAR values, the LP-80 estimates the LAI using a simplified version of the Norman-Jarvis (Norman & Jarvis, 1974) radiation transmission and scattering model. The instrument responses were validated through comparisons with a total of 21 destructive LAI samplings collected on the same dates as the other measurements. The destructive samples were collected within the experimental plots, each covering 1 m^2^ of maize plants.

In 2020, ground measurements were performed soon after the damage at the 8th-leaf stage on June 19 and were repeated on July 14, August 6 and September 16 (on which the damage at the dough stage was also performed). In 2021, ground LAI measurements were conducted 7–10 days after each damage event, synchronised with remote measurements (on June 9, July 28, and August 25), and allowing the crops to undergo their full morphologic and physiologic responses (e.g., leaf-drying and the development of necrosis). Since the flowering (“F”) and early-milky (“M”) stages were close in time, a single field survey was done on the same date, accounting for both these treatments. Surveys were conducted from mid-morning to mid-afternoon on clear-sky days, and three averaged LAI_Cept_ readings were taken at each position (Pokovai & Fodor, 2019). Each reading was calculated as an average of five individual measurements taken in different probe positions. This helped to decrease the influence of accidental leaves movements. Measurements were taken perpendicularly to crop rows, scanning an area of approximately 1 m^2^ under the canopy. Before each measurement, the external PAR sensor was used to calibrate the LP-80 probe, thus ensuring that the response between the external sensor and the probe was consistent.

### Remote sensing monitoring

In 2020, a total of 11 atmospherically corrected Sentinel-2 L2A images (hereinafter, S2) were selected as suitable (containing no visible clouds or haziness at the experimental site). The acquisition times of the images spanned through the cropping season, from June 19 to September 5. In 2021, 15 images were selected, acquired from June 2 to September 10. All images were processed using the European Space Agency (ESA) Sentinel Application Platform (SNAP) v8.0.9 (ESA, http://step.esa.int). The bands selected for the NDVI calculation were $B8A$, that is, the narrow-NIR vegetation band centred at 865 nm, and $B4$, the red band centred at 665 nm. The selection of B8A instead of the wider-NIR B8 was performed following the suggestion by Kaplan and Rozenstein (2021) that band 8A is best suited for LAI estimations given its narrower NIR window. In both years, a Sequoia multispectral sensor (Parrot, Paris, France) was mounted on a Matrice 600 Pro drone (DJI, Shenzhen, China), hereinafter called the UAV. The camera has a resolution of 1280 × 960 pixels and measured reflected radiation in four spectral regions, namely, the green (530–570 nm), red (640–680 nm), red-edge (730–740 nm) and near-infrared (NIR, 770–810 nm) ranges. The camera was calibrated prior to each flight. The ground pixel resolution was 0.04 m on average across all flights, with a 40% side overlap between flight swaths. Due to the intrinsic overlapping nature of UAV images, the swaths were processed by maintaining the mutual independence of the recorded strips in all processing steps. This prevented swath mosaicking and pixel resampling on the overlapping areas until the final index product. After that, the nearest-neighbour resampling method was adopted for image mosaicking. The UAV surveys were conducted around midday under clear-sky conditions and took approximately 45 min to complete, thus minimizing possible changes in light conditions and solar zenith angle.

In both 2020 and 2021, LAI was estimated using a parametric fractional vegetation cover (FVC) based method as proposed by Zeng et al. (2000) and adapted to LAI calculation by Ali et al. (2015). The method relies on the definition of an extinction coefficient parameter $k(\vartheta )$. For both UAV and Sentinel-2 parametric estimations, the 2020 ground measured LAI dataset was used for model parameter calibration. For 2021, UAV images and Sentinel-2 images that were closer in time to LAI ground surveys (June 9, July 29 and August 26) were then selected for validation. Following calibration on the 2020 dataset, a single sensor-specific $k(\vartheta )$ value was adopted, thus creating a suitable approach for model applications without further $k(\vartheta )$ estimations. The $k(\vartheta )$ was 0.59 for Sentinel-2 and 0.37 for UAV multispectral sensor. Further information about the method used for parametric LAI calculations from both Sentinel-2 and UAV is reported in supplementary materials.

Additionally, the Biophysical Processor Algorithm (LAI_S2_MLA_) at a 10 m resolution included in SNAP for all Sentinel-2 data was used for all available dates in 2020 and 2021. The algorithm uses a trained neural network to derive LAI values from the top-of-canopy-level reflectance and is considered to perform reasonably well over a large array of vegetation types. In the text, LAI values estimated with the parametric method are addressed as LAI_S2_, while the LAI values estimated with the machine learning algorithm included in the biophysical processor are addressed as LAI_S2_MLA_.

### Statistical analysis

The ground-measured and remote sensing-based LAI values were analysed with a linear mixed-effect model, taking the damage intensity as a fixed factor and the EC_a_ at 0–0.25 m as a continuous factor. Post-hoc pairwise comparisons of the least-square means were performed using the Tukey test (α = 0.05). The coefficient of determination (R^2^) was used to estimate the proportion of LAI_Cept_ explained by the remotely sensed LAI (LAI_S2_, LAI_S2_MLA_ and LAI_UAV_), while the root mean square error (RMSE) (1) was used to quantify the LAI estimation accuracy as follows:

1 $$RMSE = \sqrt {\mathop \sum \limits_{i = 1}^{n} \frac{{(LAI_{sim} \left( i \right) - { }LAI_{Cept} \left( i \right))^{2} }}{n}}$$

where *n* is the number of samples.

The statistical analysis was performed using the R programming language (R Core Team, 2022).

## Results

### Weather conditions during the two-year experimentation

Compared to 2020, 2021 showed lower monthly average temperatures throughout the cropping season —from April to September— particularly in April (− 2.7 °C) (Table 1). Conversely, June had higher average temperatures in 2021 (+ 1.7 °C). The absolute maximum and minimum monthly temperatures occurred in August (34.1 °C) and April (− 3.3 °C), respectively, both during 2021. The total rainfall was slightly higher in 2020 than in 2021, amounting at 353.0 mm compared to 331.0 mm, but was strongly variable when comparing the same months. Especially in June, the cumulative rainfall varied from 118.8 mm in 2020 to 6.2 mm in 2021, while more than 120.0 mm of rainfall was recorded in May 2021 vs. only 47.2 mm recorded in May 2020. No natural hailstorm events damaged the crops in either year.

**Table 1:** Temperature and rainfall data for the 2020 and the 2021 maize cropping seasons (April to September) on the experimental field

		Temperature (°C)			Rainfall (mm)
Year	Month	Average	Max	Min	Monthly total
2020	Apr	13.9	20.6	7.1	30.8
	May	17.9	23.3	12.5	47.2
	June	21.4	26.5	16.3	118.8
	July	24.0	29.8	18.3	43.2
	Aug	24.9	30.5	19.3	110.2
	Sept	21.6	27.4	15.8	2.8
2021	Apr	11.1	21.2	− 3.3	78.6
	May	15.3	23.1	5.4	123.4
	June	23.1	32.3	10.6	6.2
	July	24.1	33.3	15.1	83.4
	Aug	23.2	34.1	13.0	39.8
	Sept	20.6	28.6	12.6	0.0

### NDVI response following damage

In 2020, NDVI_S2_ and NDVI_UAV_ showed fluctuations likely related to the damage intensities. Compared to the CTRL, the highest reductions occurred in V8-Hi soon after the damage event on June 19, corresponding to -33.9% and -44.9% for S2 and UAV, respectively (Table 2). These differences decreased later in the season at the “D” stage; no reductions were observed on September 17, and even higher “Me” and “Hi” values were observed compared to the CTRL.

**Table 2:** Average NDVI values in the treatment plots measured with Sentinel-2 and UAV multispectral sensor on both 2020 and 2021

Survey Date	Time of damage	Damage intensity	NDVI_S2_	% Variation vs CTRL	NDVI_UAV_	% variation vs CTRL
19/06/2020	CTRL		0.62 ± 0.02	–	0.49 ± 0.02	–
	V8	Lo	0.58 ± 0.05	− 6.4	0.45 ± 0.02	− 8.2
		Me	0.50 ± 0.03	− 19.3	0.36 ± 0.01	− 26.5
		Hi	0.41 ± 0.04	− 33.9	0.27 ± 0.02	− 44.9
14/07/2020	CTRL		0.88 ± 0.01	–	0.83 ± 0.00	–
	V8	Lo	0.88 ± 0.00	0.0	0.81 ± 0.01	− 2.4
		Me	0.88 ± 0.00	0.0	0.80 ± 0.01	− 3.6
		Hi	0.86 ± 0.01	− 2.3	0.79 ± 0.01	− 4.8
06/08/2020	CTRL		0.89 ± 0.00	–	0.80 ± 0.01	–
	V8	Lo	0.89 ± 0.00	0.0	0.81 ± 0.00	1.3
		Me	0.89 ± 0.00	0.0	0.80 ± 0.00	0.0
		Hi	0.88 ± 0.00	− 1.1	0.79 ± 0.00	− 1.3
17/09/2020	CTRL		0.53 ± 0.03	-	–	–
	V8	Lo	0.53 ± 0.03	0.0	–	–
		Me	0.57 ± 0.02	7.6	–	–
		Hi	0.56 ± 0.02	5.7	–	–
	D	Lo	0.57 ± 0.02	7.6	–	–
		Me	0.58 ± 0.04	9.4	–	–
		Hi	0.58 ± 0.05	9.4	–	–
09/06/2021	CTRL		0.47 ± 0.00	–	0.22 ± 0.05	–
	V8	Lo	0.46 ± 0.01	− 2.6	0.27 ± 0.01	21.1
		Me	0.45 ± 0.01	− 4.5	0.24 ± 0.02	7.6
		Hi	0.44 ± 0.00	− 5.9	0.21 ± 0.00	− 3.0
28/07/2021	CTRL		0.84 ± 0.02	–	0.74 ± 0.01	–
	V8	Lo	0.83 ± 0.02	− 1.2	0.76 ± 0.00	2.4
		Me	0.81 ± 0.03	− 3.1	0.75 ± 0.00	2.2
		Hi	0.82 ± 0.02	− 1.8	0.75 ± 0.00	1.5
	F	Lo	0.80 ± 0.00	− 4.7	0.74 ± 0.00	− 0.2
		Me	0.79 ± 0.01	− 5.4	0.73 ± 0.00	− 1.5
		Hi	0.75 ± 0.01	− 11.3	0.70 ± 0.01	− 5.7
	M	Lo	0.81 ± 0.01	− 3.8	0.71 ± 0.02	− 3.6
		Me	0.78 ± 0.02	− 6.7	0.70 ± 0.03	− 5.9
		Hi	0.76 ± 0.01	− 9.2	0.69 ± 0.03	− 7.0
25/08/2021	CTRL		0.62 ± 0.03	–	0.55 ± 0.08	–
	V8	Lo	0.66 ± 0.02	7.5	0.67 ± 0.00	22.8
		Me	0.66 ± 0.01	6.7	0.66 ± 0.00	21.8
		Hi	0.62 ± 0.03	1.1	0.65 ± 0.01	19.4
	F	Lo	0.65 ± 0.01	5.9	0.60 ± 0.01	10.6
		Me	0.66 ± 0.02	7.6	0.60 ± 0.02	9.0
		Hi	0.60 ± 0.06	− 2.4	0.54 ± 0.04	− 0.3
	M	Lo	0.51 ± 0.06	− 17.6	0.47 ± 0.13	− 13.9
		Me	0.45 ± 0.06	− 26.9	0.43 ± 0.14	− 20.6
		Hi	0.47 ± 0.08	− 24.1	0.43 ± 0.14	− 21.8
	D	Lo	0.55 ± 0.08	− 10.6	0.59 ± 0.01	7.8
		Me	0.53 ± 0.08	− 14.6	0.56 ± 0.00	3.2
		Hi	0.52 ± 0.07	− 16.0	0.50 ± 0.03	− 9.2

% of variation indicates the relative variation compared to control plots (CTRL)

Compared to the CTRL, in 2021 “V8” damaged plots showed decreased NDVI_S2_ values, ranging from -2.6% in the lower-damage treatment to − 5.9% in the higher-damage. The NDVI_UAV_ values exhibited different behaviour, with the index being slightly lower than the control only in V8-Hi (− 3.0%) and higher than the control in both V8-Me (+ 7.6%) and V8-Lo (+ 21.1%). Notably, the undamaged plots were characterized by higher variability than the damaged plots. Compared to the control, the NDVI response following the “V8” damage event was maintained on July 28 for both the Sentinel-2 (Lo, − 1.2%; Me, − 3.1%; and Hi, − 1.8%) and UAV (Lo, + 2.4%; Me, + 2.2%; and Hi, + 1.5%) data. In contrast, different results were found on August 25, when both remote surveys reported higher NDVI values under damaged maize than under undamaged maize.

During the flowering (“F”) and early milky (“M”) stages, hail damage yielded NDVI reductions that agreed with the treatment intensity. NDVI was first detected on July 28, with average decreases of − 7.1% and − 6.6% in “F” and “M”, respectively, compared to the control when estimated from the Sentinel-2 data. These reductions were of − 2.4% and − 5.5% in “F” and “M”, respectively, when estimated from the UAV data. On August 25, increases in both NDVI_S2_ and NDVI_UAV_ were found in “F” compared to the control, thus confirming the previous observations on “V8”. Only the F-Hi treatment showed lower NDVI values in both the S2 (− 2.4%) and UAV (− 0.3%).

During the last survey, monitoring was also performed on maize damaged during the dough (“D”) stage. In this case, the NDVI_S2_ values were confirmed to be lower in the damaged plots than in the undamaged plots, while the NDVI_UAV_ values fluctuated between relatively high and low values. In both cases, the damaged plots showed decreased NDVI values as the damage intensity increased (Table 2).

### Ground-measured LAI dynamics

**Fig. 3:**
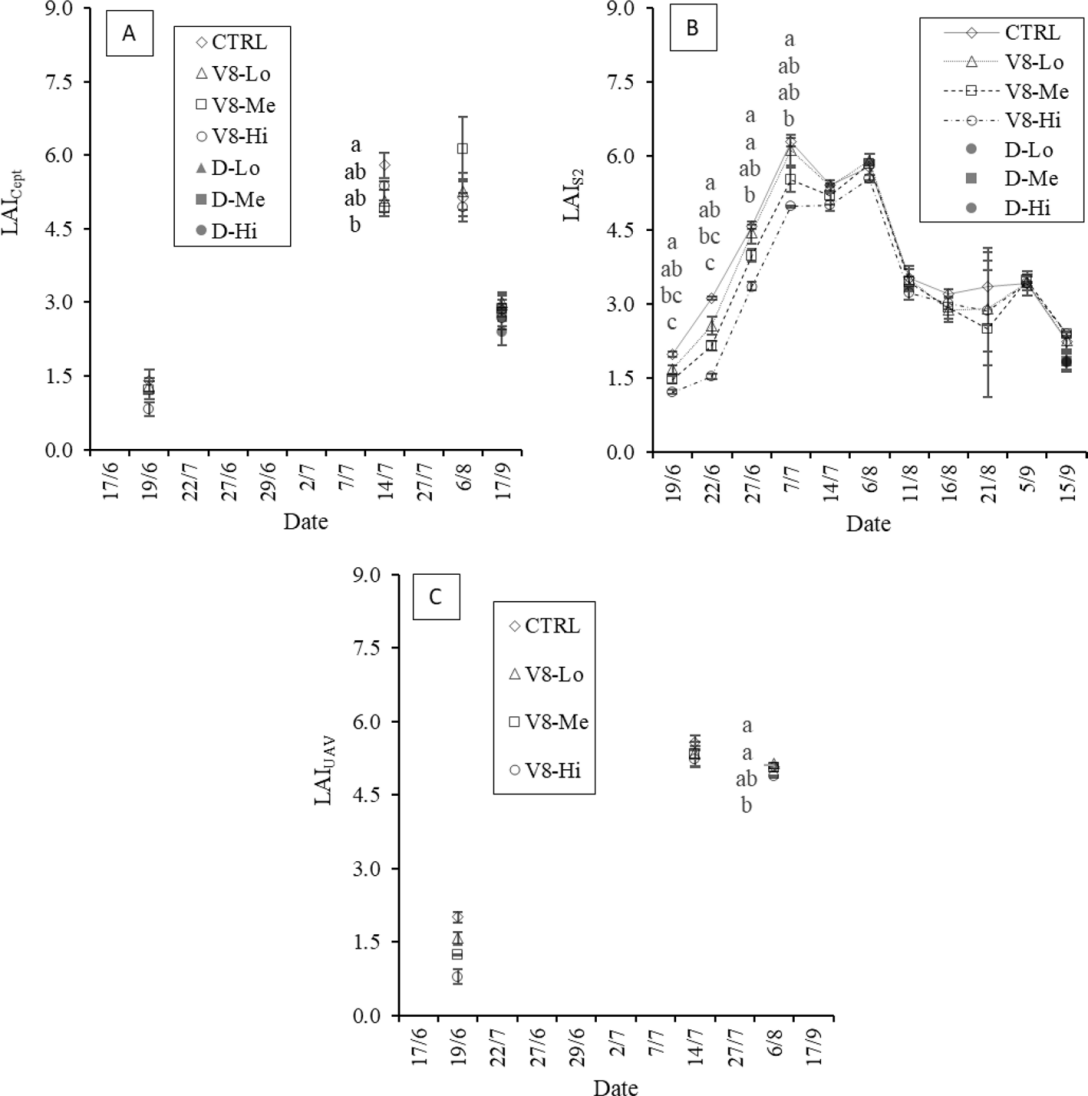
2020 LAI dynamics for ground-measured LAI (**A**), Sentinel-2-estimated LAI (**B**) and UAV-estimated LAI (**C**) at the three different damage levels and the control. For (**A**) and (**B**), the dough-stage damage is also reported (not monitored by UAV in 2020). Significant differences between treatments are highlighted (α = 0.05)

In 2020, the ground-measured LAI results (Fig. 3A) reflected a gradient with higher LAI values in the control than in the damaged plots. This trend was confirmed on July 14 but was lost on the later dates, when the differences between treatments were not significant.

**Fig. 4:**
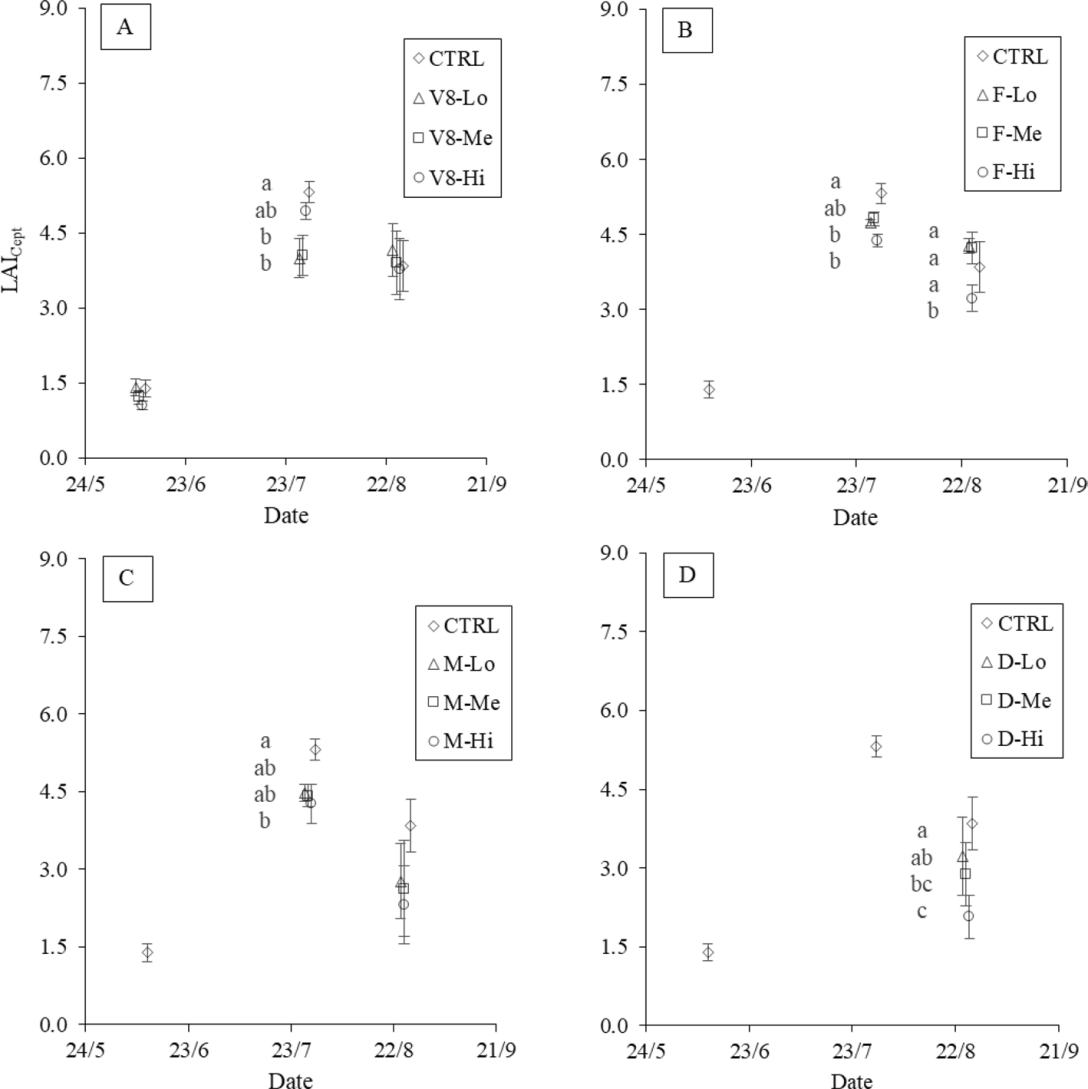
2021 LAI dynamics following damages at different plant developmental stages as measured in the field with the indirect method (LAI_Cept_). Damages occurring at the 8th-leaf (**A**), flowering (**B**), milky (**C**) and dough (**D**) stages are reported. Significant differences between treatments are highlighted (α = 0.05)

In 2021, the ground-measured LAI averaged 1.39 in the control plots during the “V8” stage (on June 9), while the damaged plots showed LAI values of 1.41, 1.22 and 1.06 in the V8-Lo, V8-Me and V8-Hi stages, respectively (Fig. 4A). On July 28 higher LAI_Cept_ values were found in the control (5.32) than in the “V8” damaged plots (4.33). However, among damaged treatments, no reduction corresponding to increasing damage intensities was evident. A further lack of differentiation between the treatment and control plots was observed on the last sampling date (August 25). During the “F” stage (Fig. 4B), LAI reductions following damage were evident, and the F-Hi and control plots had the lowest and highest LAI values, respectively (4.38 and 5.32). On the last survey date, this differentiation was not significant, with the control being lower than the F-Lo and F-Me values (3.85 versus 4.28 and 4.23, respectively). When the damage occurred at the “M” stage, a similar differentiation pattern as that of the “F” stage was observed on July 28, with the control being significantly different from the “Hi” treatment (Fig. 4C). The field surveys following the damage that occurred during the “D” stage were performed only on August 25 (Fig. 4D). The LAI values obtained showed decreasing values compared to the control as the damage intensity increased, with indexes of 3.22, 2.88 and 2.07 in the D-Lo, D-Me and D-Hi treatments, respectively.

### Calibration and validation of remote sensing LAI estimates

**Fig. 5:**
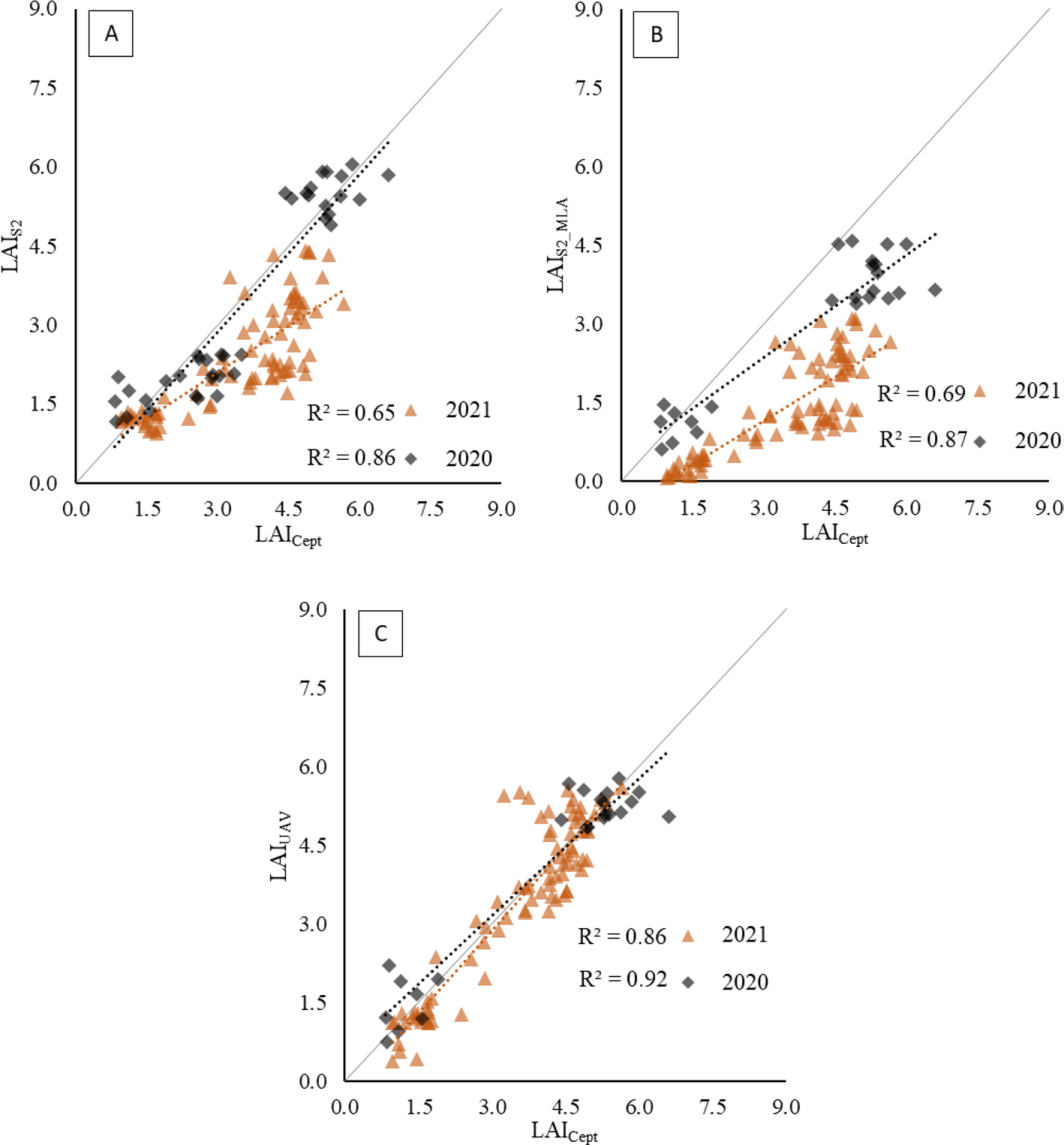
Regression results between the Sentinel-2 parametrically estimated (**A**) LAI_S2_, Sentinel-2 machine-learning-estimated LAI_S2_MLA_ (**B**) and UAV-estimated LAI_UAV_ (**C**) and the ground measured LAI_Cept_ for both 2020 (calibration) and 2021 (validation). In the case of LAI_S2_MLA_, only the estimation process is reported

Validation of the remote-sensed LAI measurements was performed during 2021 based on calibration parameters obtained during the 2020 cropping season and reported in Table 3. In both 2020 and 2021, the LAI values estimated using NDVI_S2_ (LAI_S2_) showed good agreement with the ground-based measurements, with 86% of the total variability explained in 2020 and 65% explained in 2021 (Fig. 5A). However, in 2021, LAI_S2_ generally underestimated the ground-based values along the entire range of measured LAI, leading to a RMSE of 1.37 (Table 3). The LAI values estimated using the Sentinel-2 biophysical processor (LAI_S2_MLA_) were not calibrated; therefore, both 2020 and 2021 values were used to evaluate the goodness of fit of the machine learning data compared with the ground data (Fig. 5B). The results showed a slightly better R^2^ than that obtained from the LAI_S2_ values, but the ground-measured LAI was systematically underestimated. In particular, this situation was observed for 2021, with the LAI_S2_MLA_ values being almost halved compared to the LAI_Cept_ values. Conversely, better agreement was found between LAI_UAV_ and LAI_Cept_ in both 2020 (R^2^ of 0.92, RMSE of 0.10) and 2021 (R^2^ of 0.86, RMSE of 0.75), despite the slight underestimations observed in 2021 and the slightly better agreement observed at relatively high LAI values in 2020 (Fig. 5C).

**Table 3:** Calibration (2020) and validation (2021) parameters for LAI_S2_ and LAI_UAV_ estimation

		2020		2021	
	k(ϑ)	R^2^	RMSE	R^2^	RMSE
LAI_S2_	0.59	0.86	0.68	0.65	1.37
LAI_S2_MLA_	n.d	0.87	1.30	0.69	2.19
LAI_UAV_	0.37	0.92	0.10	0.86	0.71

### LAI estimation from Sentinel-2

**Fig. 6:**
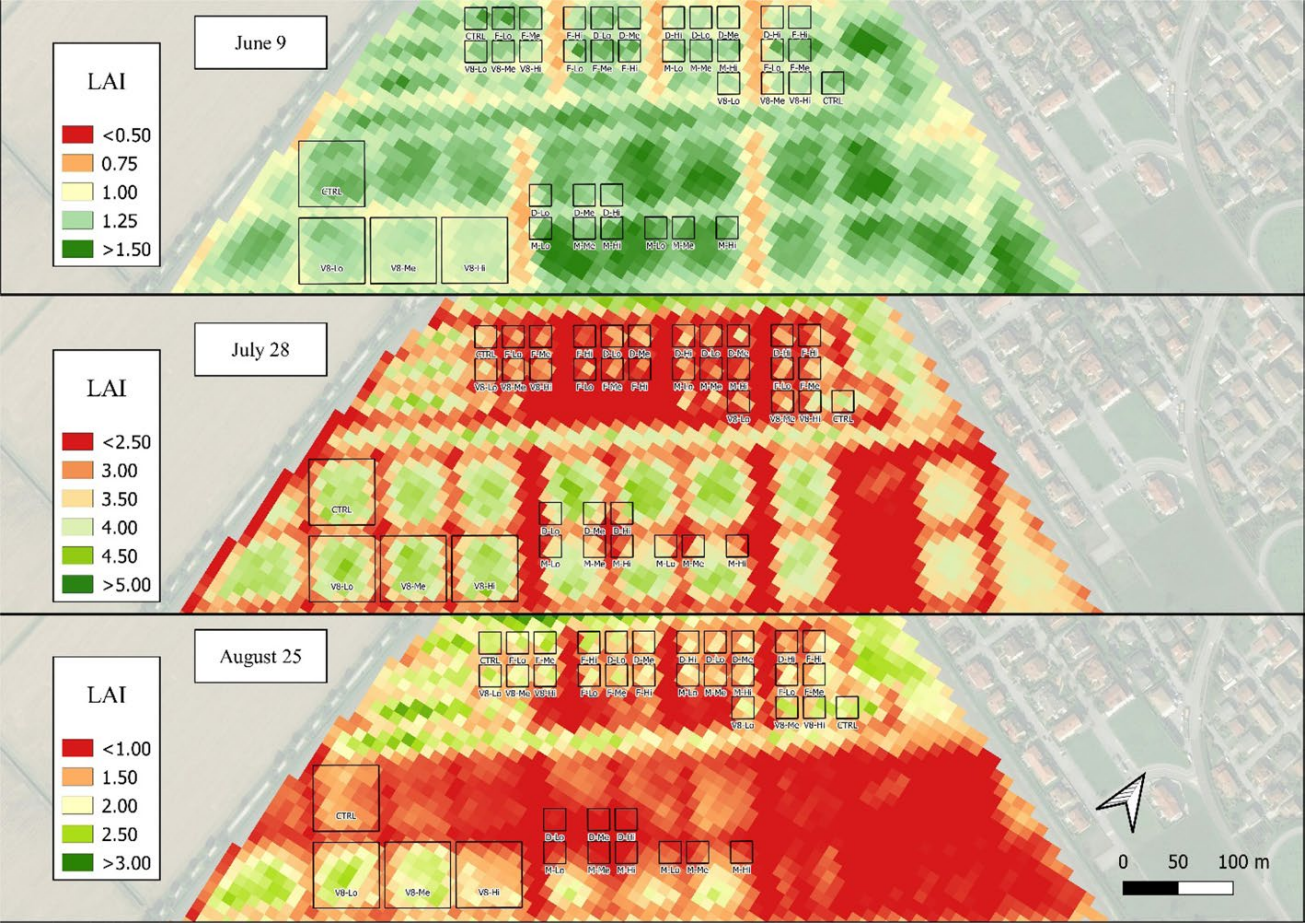
2021 map of estimated LAI_S2_ values over the experimental field for the three sampling dates of June 9, July 28 and August 25. Due to the different LAI ranges throughout the season, each date is presented with a specific legend

**Fig. 7:**
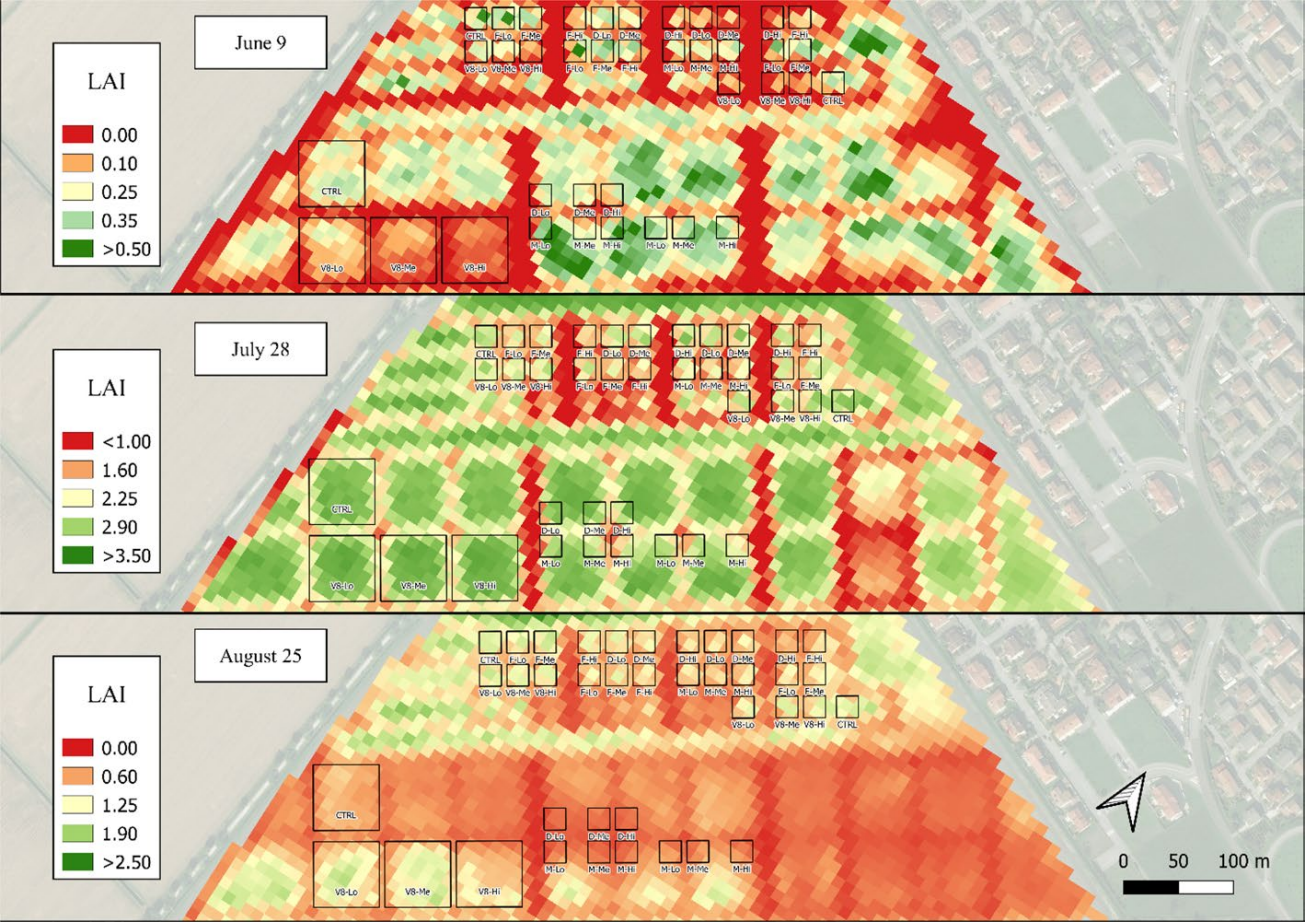
2021 map of estimated LAI_S2_MLA_ values over the experimental field for the three sampling dates of June 9, July 28 and August 25. Due to the different LAI ranges throughout the season, each date is presented with a specific legend

The estimated LAI_S2_ and LAI_S2_MLA_ maps in 2021 provided a general overview of the most-damaged areas (Figs. 6 and 7), e.g., in the “V8” plots soon after the damage occurred (June 9) and in the larger plots on August 25 (60 × 60 m). Despite the generally lower LAI_S2_MLA_ values in these maps, the observed spatial patterns tended to coincide between the two methods. LAI_S2_ maps for 2020 are reported in Fig. 1-SP in the supplementary materials. In 2020, the LAI_S2_ values estimated for the “V8” damaged stage (Fig. 3B) showed a dynamic consistent with that of the damage intensity immediately following the damage event (June 17) and lasting until mid-July. The LAI values peaked on July 7 (6.12). On the following dates, the differentiation between CTRL and damage treatments was lost, particularly from July 14 onwards, when the “Lo” and “Me” intensities reached control values. The damage done during the “D” stage led to a differentiation mainly between control and damage, but no clear gradient was visible within the damage intensities.

**Fig. 8:**
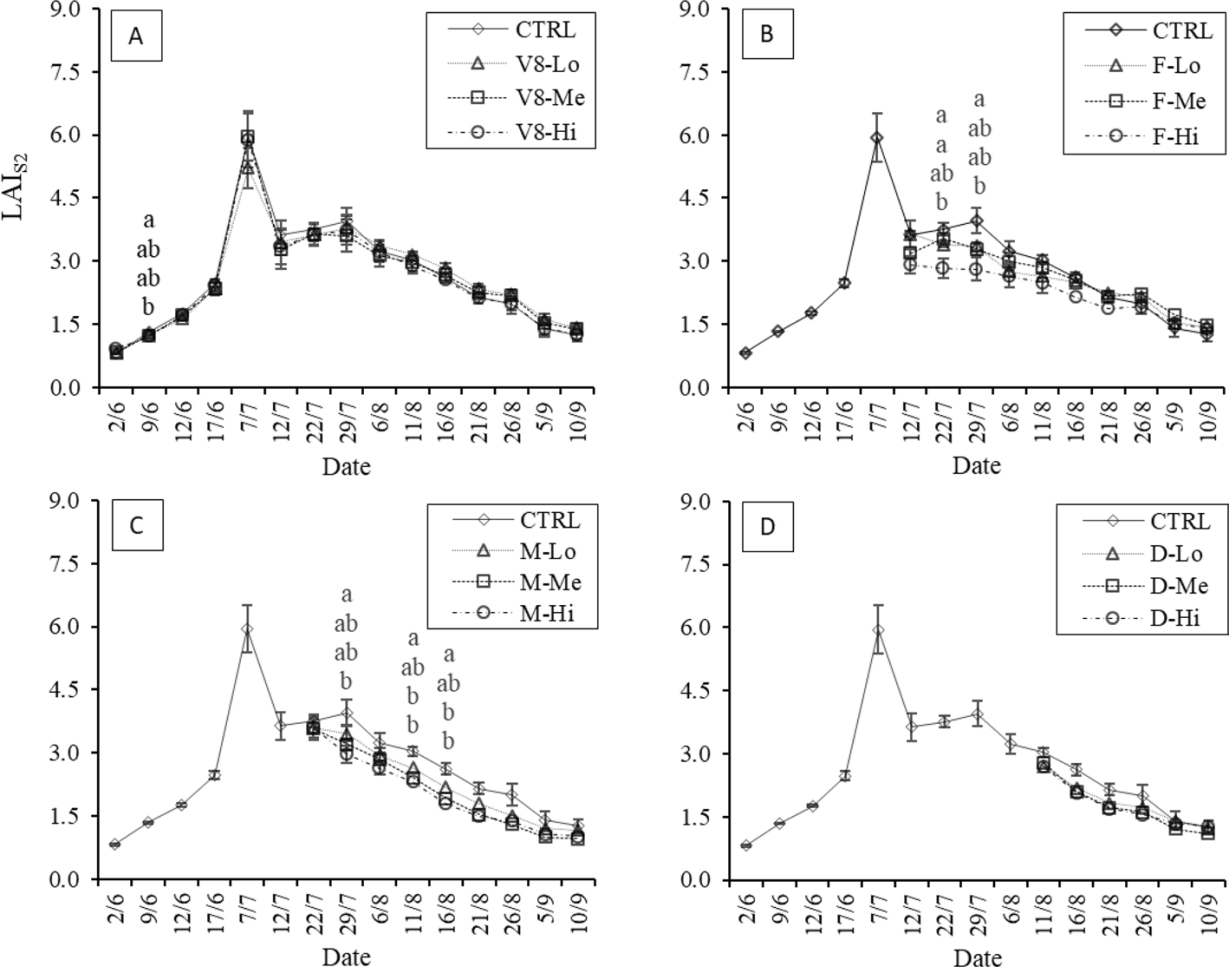
2021 temporal LAI_S2_ dynamics following damage at each stage: 8th-leaf (“V8”, image A), flowering (“F”, image B), early milky (“M”, image C) and dough (“D”, image D) stages, estimated using all available 2021 Sentinel-2 data. Significant differences between treatments are highlighted (α = 0.05)

**Fig. 9:**
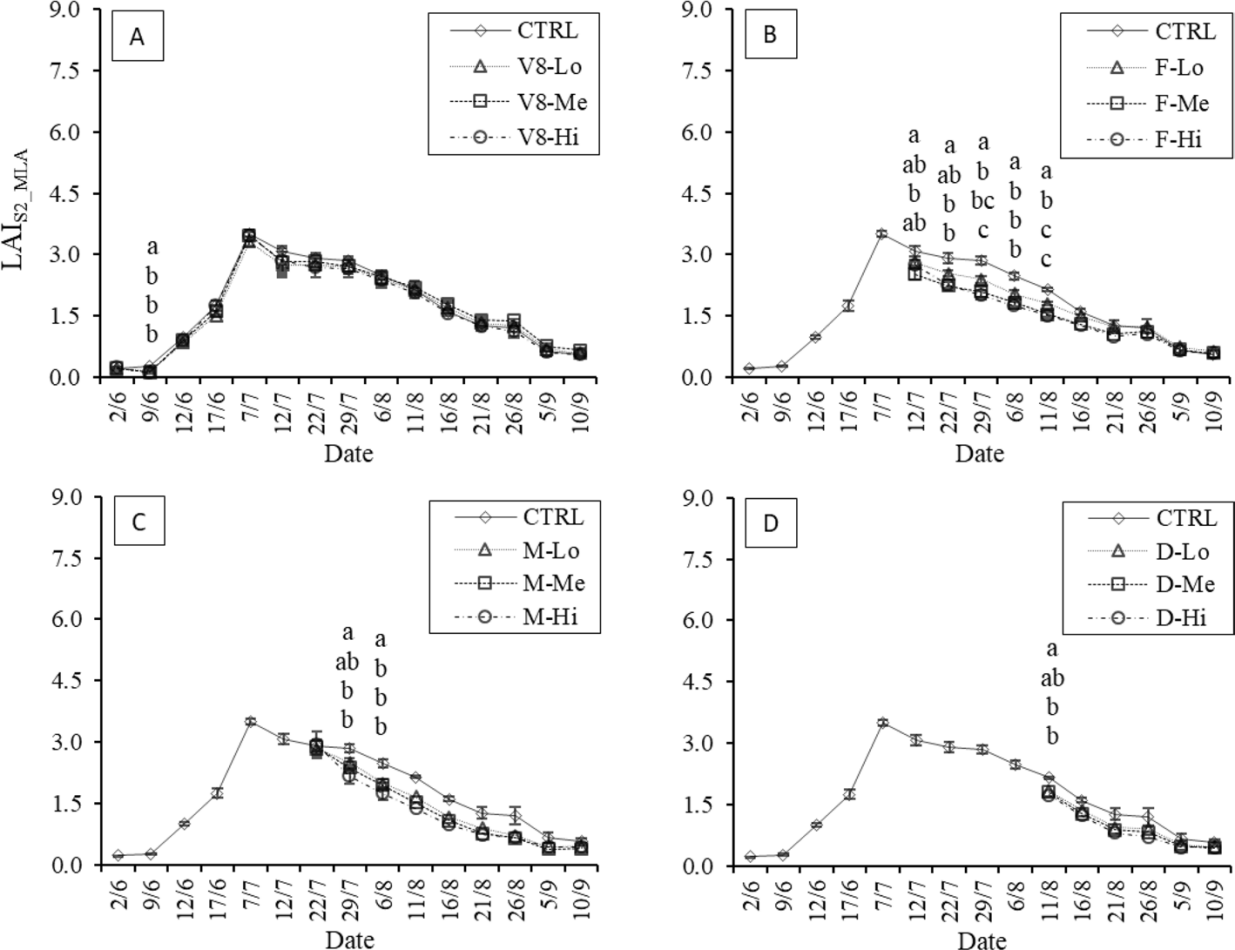
2021 temporal LAI_S2_MLA_ dynamics following damage at each stage: 8th-leaf (“V8”, image A), flowering (“F”, image B), early milky (“M”, image C) and dough (“D”, image D) stages, estimated using all available 2021 Sentinel-2 data. Significant differences between treatments are highlighted (α = 0.05)

In 2021, the estimated LAI_S2_ values increased rapidly after June 9, when the average value was approximately 1.00, until a maximum value occurred on July 7 (LAI_S2_ = 5.94) in the control, just before flowering. Most of the differences were observed between undamaged and damaged maize plots, especially at high LAI values. For instance, when damage was done during the “V8” stage (Fig. 8A), a slightly consistent gradient corresponding to the damage intensity was observed after the damage event on June 9, with a maximum decrease of -9.0% in the high-damage plots. The maximum average difference from the control was observed on July 7, reaching a reduction of -12.4%, while the minimum difference appeared on September 5 at − 0.2%; at this time, all treatments had comparable LAI values. Similarly, in the “F”, “M”, and “D” stages (Fig. 8B, C, D), most of the reductions were observed soon after damage occurred, peaking at − 28.8% in the “F” stage on July 29 in the “Hi” damaged plots. The “M” treatments showed the largest LAI differences later, on August 25, reaching -35.1%. After that, the difference was reduced steadily as LAI decreased until the end of the cropping season, with the steepest reductions observed in the “F” plots, where the control and “Lo-Me-Hi” treatments sooner converged to similar LAI_S2_ values. The “Hi”-intensity damage showed generally lower LAI_S2_ values than the other treatments. This effect was observed especially during the “F” stage, with LAI_S2_ values being consistently lower than the control values as well as than the “Lo” and “Me” treatments (Fig. 8B). Overall, the estimated LAI_S2_ values appeared to show different responses when damage was conducted at different maize developmental stages. LAI_S2_MLA_ showed a similar pattern to the Sentinel-2 parametric LAI values, highlighting the generally lower values measured across all treatments and phenological phases (Fig. 9). In particular, the evident LAI peak observed on July 7 in LAI_S2_ was less pronounced in these results. Moreover, as observed in the LAI_S2_ values, damage during the “V8” stage had less of an impact on LAI during the rest of the season than damage occurring during other stages (Fig. 9A). This resulted in little to no differentiation between treatments. Conversely, the treatment differences associated with damages done during the “F”, “M” and “D” stages were slightly better captured by the LAI_S2_MLA_ results than by the parametric method (Fig. 9B–D).

### LAI estimation from UAV sensor

**Fig. 10:**
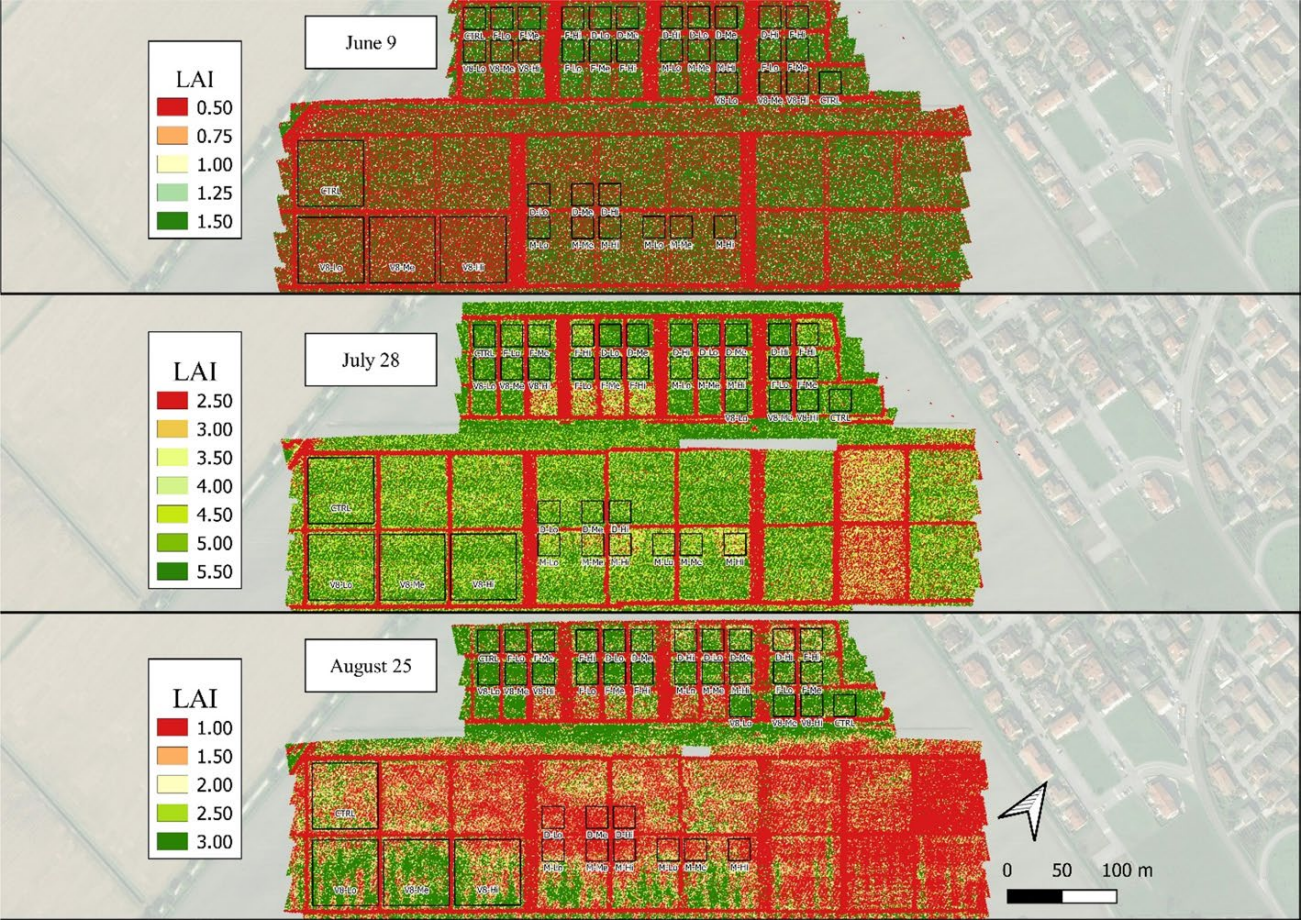
2021 map of estimated LAI_UAV_ values over the experimental field for the three sampling dates of June 9, July 28 and August 25. Due to the different LAI ranges throughout the season, each date is presented with a specific legend

The high resolution of the UAV data allowed to capture the LAI heterogeneity both between and within plots in 2021, as shown in the LAI maps (Fig. 10). For instance, in the last survey (August 25), non-homogeneous LAI conditions were found mostly in the southern part of the field. The 2020 LAI maps are reported in Fig. 2-SP in the supplementary materials. In 2020 (Fig. 3C), a clear LAI gradient among the damage intensities was visible during the “V8” stage, ranging from − 21.4% at the lower-intensity treatment to − 60.7% at the higher-intensity compared to the control (LAI of 2.01). Later in the season, this trend was lost, particularly on August 6, when the LAI values were comparable among the control and the three damage intensities.

**Fig. 11:**
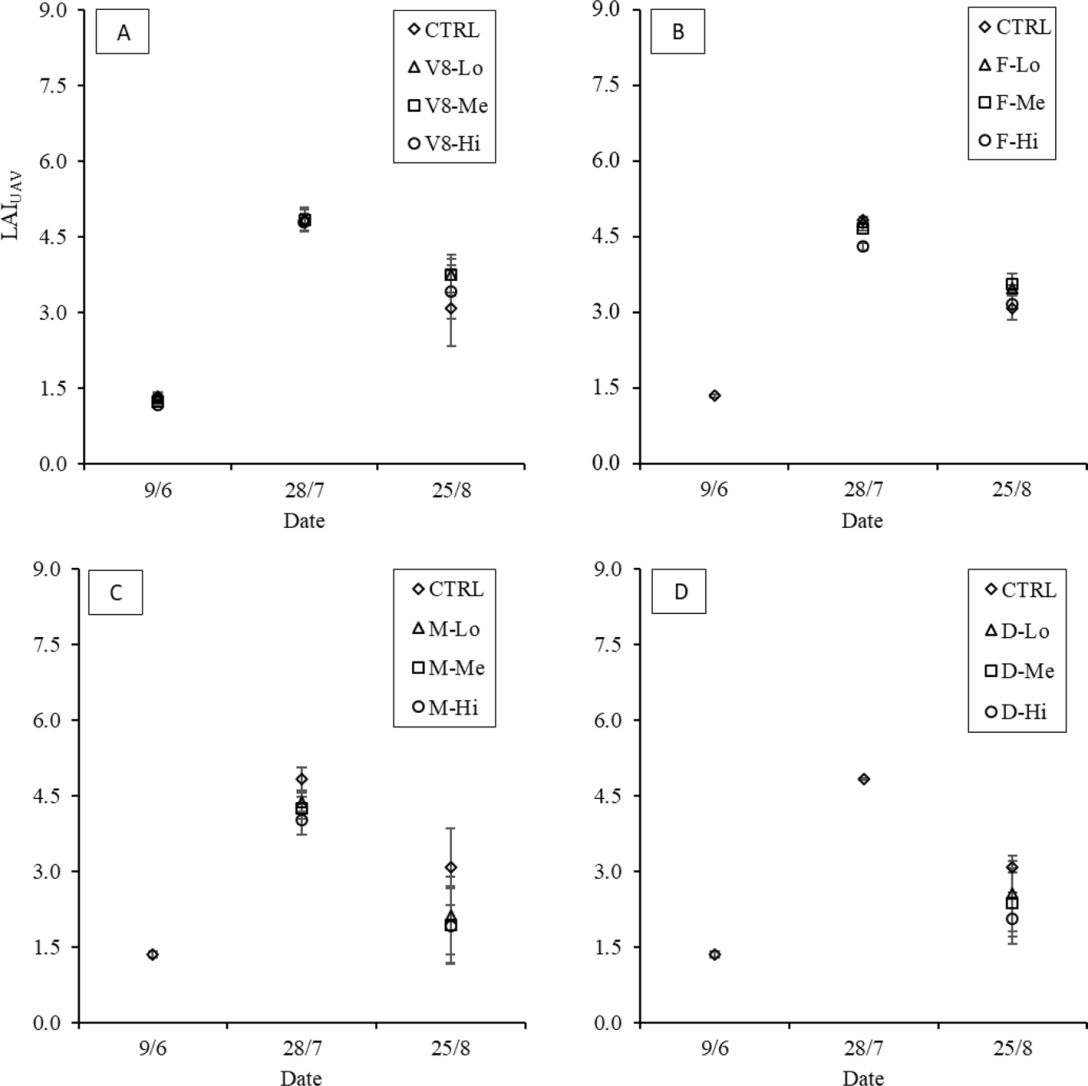
2021 temporal dynamics following damage at each stage: 8th-leaf (V8, image A), flowering (F, image B), early milky (M, image C) and dough (D, image D) stages, estimated using the data recorded by the UAV multispectral sensor. Significant differences between treatments are highlighted (α = 0.05)

In 2021, the LAI values estimated from the UAV data showed similar behaviour as the LAI_S2_ values, with relatively low values at the beginning of the season, peaking on July 28 in the control and finally decreasing on August 25 (Fig. 11). In the “V8” treatments, LAI_UAV_ showed a consistent trend immediately following the damage event on June 9 (Fig. 11A), with V8-Hi showing the greatest reduction compared to the control (− 25%). On July 28, no significant differences were observed among treatments, while the control showed lower values than the damaged plots on August 25 (-20% compared to “Me”). In “F”, the greatest difference occurred on July 28, when a LAI_UAV_ reduction was observed in F-Hi compared to the control (− 11%). In contrast, on August 25, a similar behaviour to “V8” was observed, with the control showing the lowest value, reaching -16% compared to F-Me (Fig. 11B). The LAI values measured on the “M” treatment consistently corresponded to the damage gradient on both July 28 and August 25 (Fig. 11C), with M-Hi showing the highest decreases of − 19% and − 37%, respectively, compared to the control. The lowest reductions were observed at the lowest damage intensity, with decreases of − 10% and − 30% on July 28 and August 25, respectively. Similarly, the “D” damaged plots reported the lowest “Hi” intensity values, with a decrease of − 37% compared to the control (Fig. 11D). Overall, consistent behaviour was generally observed following damage, with the notable exception of the “F” treatments, where little differentiation was generally observed, particularly on August 25.

## Discussion

In the present study, the use of remote sensing-based NDVI has shown to allow for an effective estimation of LAI in both undamaged and hail-damaged maize. Validation was conducted on a different cropping season than calibration and also included a wider range of phenological stages. This validation gave robustness to the method, which, in 2021, proved effective in estimating LAI even when the calibration was run on a 2020 dataset that included relatively few phenological stages. Moreover, the methodology was tested during both years using two sensors that collected data at different spatial resolutions, emphasizing good agreement with the ground-based measurements in both cases. Both sensors retrieved LAI reductions that corresponded with the damage intensity, consistently with ground measurements. However, the better accuracy of the LAI_UAV_ compared to the LAI_S2_MLA_ and LAI_S2_ estimations was likely due to the higher spatial resolution of the former measurement source, which could better account for small-scale variabilities at various damage levels and canopy conditions. Similar conditions have been reported by Khaliq et al. (2019) in vineyards, with the vigour assessment of vegetation biased by the low satellite resolution, which included interrow terrain pixels. Moreover, it is likely that the small-scale reflectance changes that occurred in heterogeneously damaged canopies were not fully revealed by the satellite imagery.

Recent studies (e.g., Hosseini et al., 2021) obtained even better results than ours when estimating LAI in maize by using water cloud or support vector machine models. However, the authors of those studies did not test their methods under damaged maize conditions, and they also warned that the proposed methodology still needed some implementation exploration, e.g., calibrations were conducted for three LAI intervals, and additional data other than LAI might be needed. In contrast, the robustness of this parametric approach was based on the calibration of a single crop-sensor light extinction coefficient *k*(ϑ) for the whole season rather than being dependent on phenological phases (Brogi et al., 2020). At the satellite level, the parametric method proved to be effective even when compared against Sentinel-2 Biophysical Processor LAI estimations, which are often used due to their independence from specific information regarding the field conditions and crop types (Levitan et al., 2019). The comparison between the ground-measured LAI and parametric Sentinel-2 and UAV LAI estimations showed clear underestimations in the Sentinel-2 LAI_MLA_-generated product, as was also pointed out by Djamai et al. (2019) for a variety of crops and by Xie et al. (2019) for winter wheat; however, this result contrasted with that reported by (Yu et al., 2021) for maize, where the Sentinel-2 LAI_MLA_ product resulted in fairly good estimations. Nonetheless, the MLA method was able to retrieve relative LAI reductions between undamaged and damaged vegetation consistently with those found through the parametric estimation.

Globally, these methods proved suitable for extensive remote sensing applications in which little information about the crop variety or management practices are available (Zhang et al., 2014), although the higher-resolution data provided by the UAV system led to the most accurate parametric LAI estimations, including cases where maize underwent canopy geometry changes due to the occurrence of hail events. One of the key aspects of this multispectral approach relies on its extensive applicability; even if hyperspectral methods are capable of providing full spectrum analyses, parametric multispectral approaches might overcome some currently known problems regarding the practicality of these sensors, namely, their accessibility (e.g., costs) and sophisticated data-acquisition and postprocessing steps that limit their use apart from research applications (Adão et al., 2017). Nonetheless, the use of LAI in actual yield damage assessments still needs to be explored, given that the link between LAI and yield damage can be nonlinear (Lauer et al., 2004). Moreover, crop yields can be affected by numerous factors, including pests and environmental conditions such as droughts, and these factors can also cause a drop in NDVI, thus influencing LAI retrieval. Therefore, care must be taken when linking LAI decreases and actual hailstorm events during estimations.

Moreover, $k(\vartheta )$ can be sensor-dependent when estimated from spectral data, which restricts its extensive use in LAI estimation tasks (e.g. Ali et al., 2015; Brogi et al., 2020; Zhang et al., 2014). Indeed, $k(\vartheta )$ is inverted from LAI based on VIs (e.g., NDVI) and depends on a combination of factors, such as the sensor configuration (Khaliq et al., 2019) and viewing conditions (Song et al., 2016). For instance, Song et al. (2016) reported that the canopy reflectance spectra in different observation planes changed irregularly among the spectral regions, resulting in differences in the spectral indices used for vegetation monitoring. This is also the case for field surveys conducted with Sentinel-2 and UAV data, the acquisition durations of which are inherently different and only partially synchronized (Abdelbaki et al., 2021).

Nonetheless, NDVI proved to be a good proxy for estimating LAI despite its tendency to become saturated during the later stages of the growing season. Indeed, defoliation was inversely correlated with NDVI by exposing larger areas of bare soils to the sensors, thus increasing the reflectance in the red wavelengths and decreasing the reflectance in the NIR range (Peters et al., 2000). Moreover, canopy factors such as the canopy density and clumping (Fang et al., 2019; Gitelson et al., 2014; Trenholm et al., 1999) were also affected by the simulated hailstorm damage. This led to decreases in NDVI values due to defoliation that were sufficiently large, allowing the NDVI values to fall below the saturation level (Olsson et al., 2016).

Spatially, the LAI-generated maps highlighted considerable variability within the studied fields, thus emphasizing the importance of precise spatial assessments to account for such differences. The survey timing was a critical factor in describing LAI dynamics. The 8th-leaf was found to be the stage in which maize was the least affected by damage over time due to the emission of new leaves, thus making the damages hardly detectable from remote sensing, as was previously reported by Furlanetto et al. (2021) and Peters et al. (2000) in maize and by Zhou et al. (2016) in potato crops. Conversely, damages in later stages—e.g., in the flowering, milky and dough stages—resulted in detectable differences throughout the rest of the cropping season, with the plants being unable to mask the damages (e.g., defoliation or necrosis) over time.

## Conclusions

In this work, remotely sensed NDVI values from UAV and Sentinel-2 were viable for estimating LAI in both undamaged and damaged maize plots, serving as a robust and objective approach for assessing hail damage both spatially and temporally. Spatially, the sensors were able to capture the field damage, despite a different degree of accuracy. In fact, the UAV-based surveys proved to be the most accurate, possibly resolving the microscale canopy geometry and background detection conditions that were pivotal for obtaining accurate LAI estimations. This advantage came at the cost of a limited monitoring area and temporally demanding planning. This effect was especially observed when damage occurred at the early growth stages of maize, after which rapid vegetation recovery could mask defoliation and make damage detection harder, thus requiring prompt monitoring. Regarding timing, LAI was effectively modelled in all phenological phases, even under full canopy closure. NDVI saturation was here not a limiting factor, possibly due to the increased exposure of bare soil following defoliation. Parametric multispectral approaches might also overcome some currently known problems regarding the practicality of hyperspectral sensors. Moreover, a single value of extinction coefficient $k(\vartheta )$ was adopted regardless of the growing phase, thus simplifying the adoption of specific parameters based on the phenological phase of the crops. When compared against Sentinel-2 Biophysical Processor LAI, the parametric approach was effective, notably leading to less underestimation and a slightly better accuracy when estimated using the same Sentinel-2 data. The UAV approach proved to be the most accurate overall. It follows that this methodology could be applied to study maize throughout the cropping season. Nevertheless, the $k(\vartheta )$ value was sensor dependent, suggesting that a separate calibration step could be needed. Future research should refine the application of this methodology at the coarser satellite ground resolutions. Finally, further investigations should be conducted to test and validate this parametric method on other crops and on a wider range of damage intensities.

## Supplementary Information

Below is the link to the electronic supplementary material.

Supplementary file1 (DOCX 3281 KB)

## Data Availability

The datasets generated during and/or analysed during the current study are not publicly available.
