# Supplementary material for: LAI estimation through remotely sensed NDVI following hail defoliation in maize (Zea mays L.) using Sentinel-2 and UAV imagery
Source: Precis Agric. 2023 Feb 27:1–25. Online ahead of print. doi: 10.1007/s11119-023-09993-9 (PMC9968646; doi:10.1007/s11119-023-09993-9)
Supplement: Supplementary file 1 — Supplementary file1 (DOCX 3281 KB) [file 11119_2023_9993_MOESM1_ESM.docx]

LAI estimation through remotely sensed NDVI following hail defoliation in maize (*Zea mays* L.) using Sentinel-2 and UAV imagery.

J. Furlanetto^1*^, N. Dal Ferro^2^, M. Longo^2^, L. Sartori^1^, R. Polese^2^, D. Caceffo^3^, L. Nicoli^3^, F. Morari^2^

*^1^University of Padova, TESAF dept., Legnaro, 35020, Padova, Italy,*

*^2^University of Padova, DAFNAE dept., Legnaro, 35020, Padova, Italy,*

*^3^Società Cattolica di Assicurazione S.C., Verona, 37126, Italy,*

*corresponding author: jacopo.furlanetto@phd.unipd.it

SUPPLEMENTARY MATERIAL

LAI estimation from remote sensing

In both years, LAI was estimated using a parametric fractional vegetation cover (FVC) based method as proposed by Zeng et al. (2000) and adapted to LAI calculation by Ali et al. (2015). For Sentinel-2 images, NDVI (hereafter NDVI_S2_) was calculated as (1):

${NDVI}_{S2}=\frac{B8A - B4}{B8A + B4}$, (1)

where $B8A$ is the vegetation narrower-NIR band centred at 865 nm and $B4$ is the red band centred at 665 nm. NDVI was calculated from the UAV-borne sensor (hereafter NDVI_UAV_) as follows (2):

${NDVI}_{UAV}=\frac{\rho_{790} - \rho_{660}}{\rho_{790}+ \rho_{660}}$, (2)

where $\rho_{790}$ indicates the NIR central wavelength reflectance and $\rho_{660}$ the red central wavelength reflectance.

The FVC was therefore calculated as (1):

$FVC=\frac{NDVI- {NDVI}_{s}}{{NDVI}_{V}- {NDVI}_{s}}$ (1)

for both UAV- and S2-based NDVI values. NDVI_s_ represents the NDVI value for bare soil (-0.10) and NDVI_v_ represents the NDVI value at full vegetation cover amongst all available images (either UAV or S2), i.e., a pure pixel representing only vegetation. Images were masked for the single experimental plots, thus avoiding to include other crop species in the scene. Sentinel-2 NDVI_v_ value was set as 0.94, whereas UAV NDVI_v_ as 0.92.

FVC was then used for LAI calculation as in equation (2) (Ali et al., 2015):

$LAI=\frac{- ln(1 - FVC)}{k(\vartheta)}$ (2)

where $k(\vartheta)$ is the light extinction coefficient, defined as in equation (3):

$k(\vartheta)=\frac{- ln(\frac{I}{I_{o}})}{LAI}$ , (3)

where $I$ is the transmitted radiation through the canopy and $I_{o}$ is the incoming radiation (above the canopy). The $\frac{I}{I_{o}}$ ratio can be defined as $1-FVC$ as in equation (4) (Propastin & Erasmi, 2010). The $k(\vartheta)$ accounts for light attenuation in the canopy and is mainly dependent on the canopy structure and the solar zenith angle. Radiation reaching the ground from the top of the canopy decreases as $k(\vartheta)$ increases. For a given LAI, $k(\vartheta)$ accounts for changes in the canopy structure among different plant species (Campbell, 1986; Monsi et al., 2005) and, in the same species, for the different canopy structure during the plant growing cycle, e.g., due to azimuthal leaf orientation (Drouet & Moulia, 1997).

**References**

Ali, M., Montzka, C., Stadler, A., Menz, G., Thonfeld, F., & Vereecken, H. (2015). Estimation and validation of RapidEye-based time-series of Leaf Area Index for winter wheat in the Rur catchment (Germany). *Remote Sensing*, *7*(3), 2808–2831. https://doi.org/10.3390/rs70302808

Campbell, G. S. (1986). Extinction coefficients for radiation in plant canopies calculated using an ellipsoidal inclination angle distribution. *Agricultural and Forest Meteorology*, *36*(4), 317–321. https://doi.org/10.1016/0168-1923(86)90010-9

Drouet, J. L., & Moulia, B. (1997). Spatial re-orientation of maize leaves affected by initial plant orientation and density. *Agricultural and Forest Meteorology*, *88*(1–4), 85–100. https://doi.org/10.1016/S0168-1923(97)00047-6

Monsi, M., Saeki, T., & Schortemeyer, M. (2005). On the factor light in plant communities and its importance for matter production. *Annals of Botany*, *95*(3), 549–567. https://doi.org/10.1093/aob/mci052

Propastin, P., & Erasmi, S. (2010). A physically based approach to model LAI from MODIS 250m data in a tropical region. *International Journal of Applied Earth Observation and Geoinformation*, *12*(1), 47–59. https://doi.org/10.1016/j.jag.2009.09.013

Zeng, X., Dickinson, R. E., Walker, A., Shaikh, M., Defries, R. S., & Qi, J. (2000). Derivation and evaluation of global 1-km fractional vegetation cover data for land modeling. *Journal of Applied Meteorology*, *39*(6), 826–839. https://doi.org/10.1175/1520-


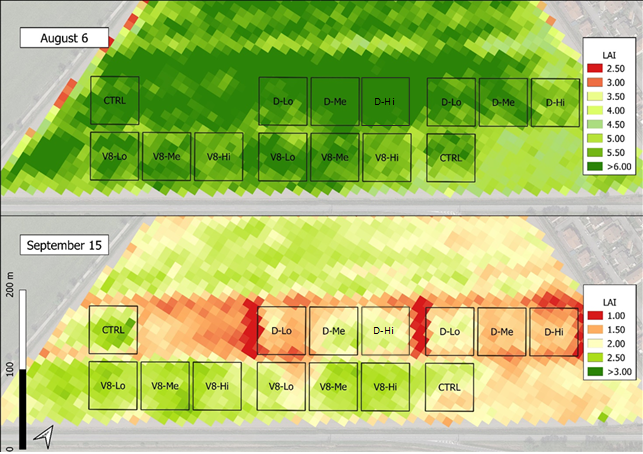

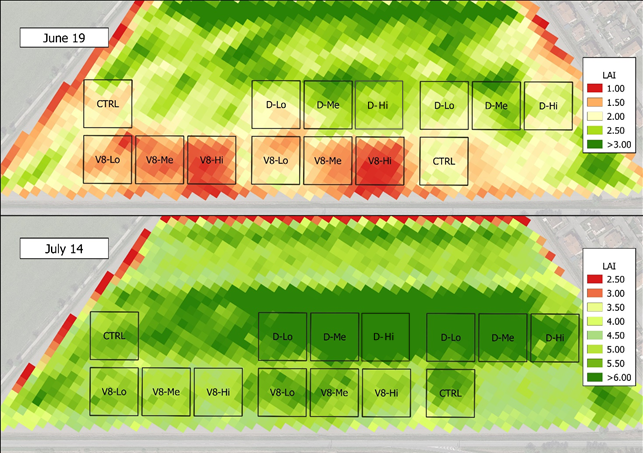


**Figure 1-SP**: Estimated LAI_S2_ map over the experimental field for the four sampling dates of June 19, July 14, August 6 and September 15. Due to different LAI ranges across the season, every date is presented with a specific legend.


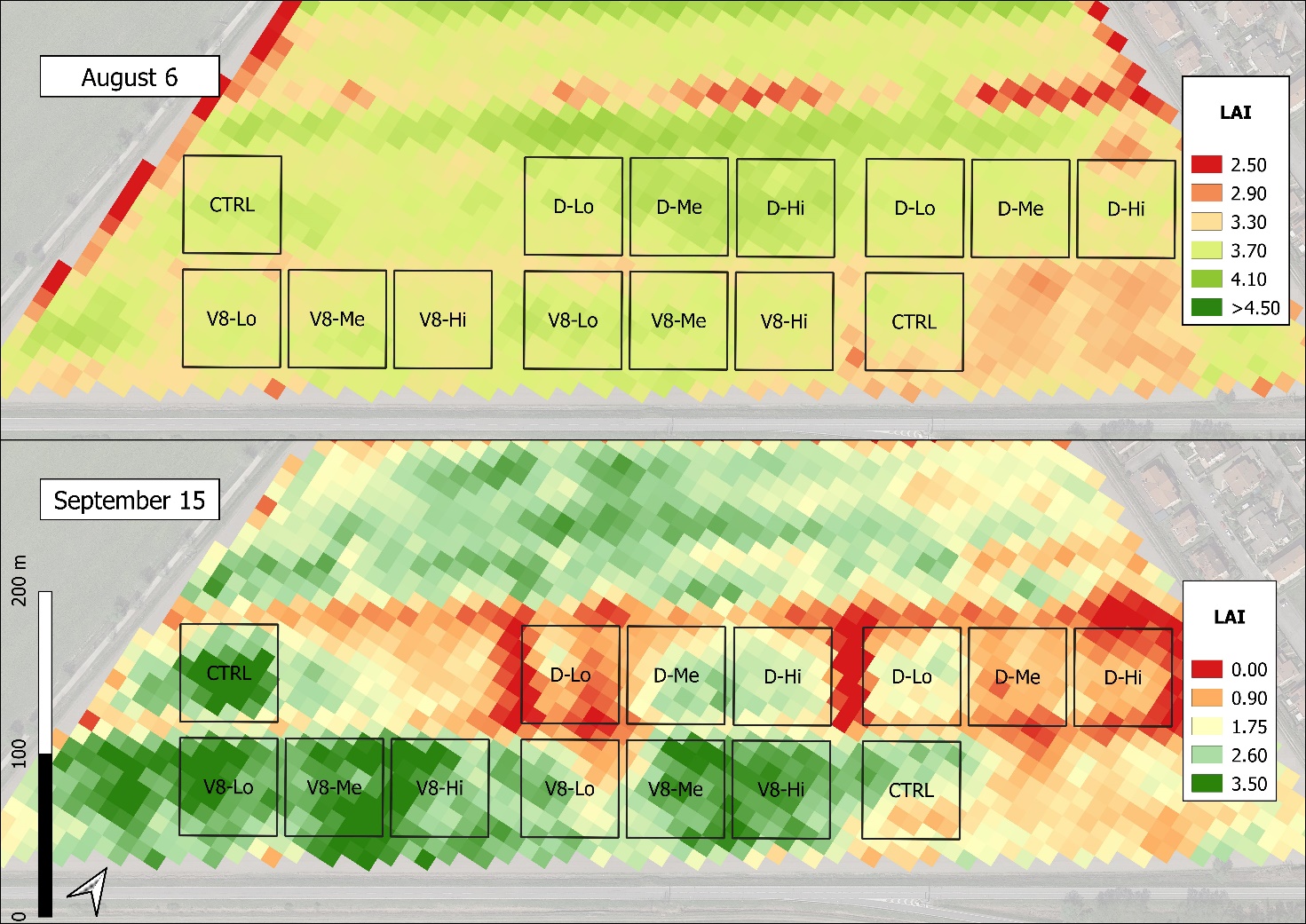

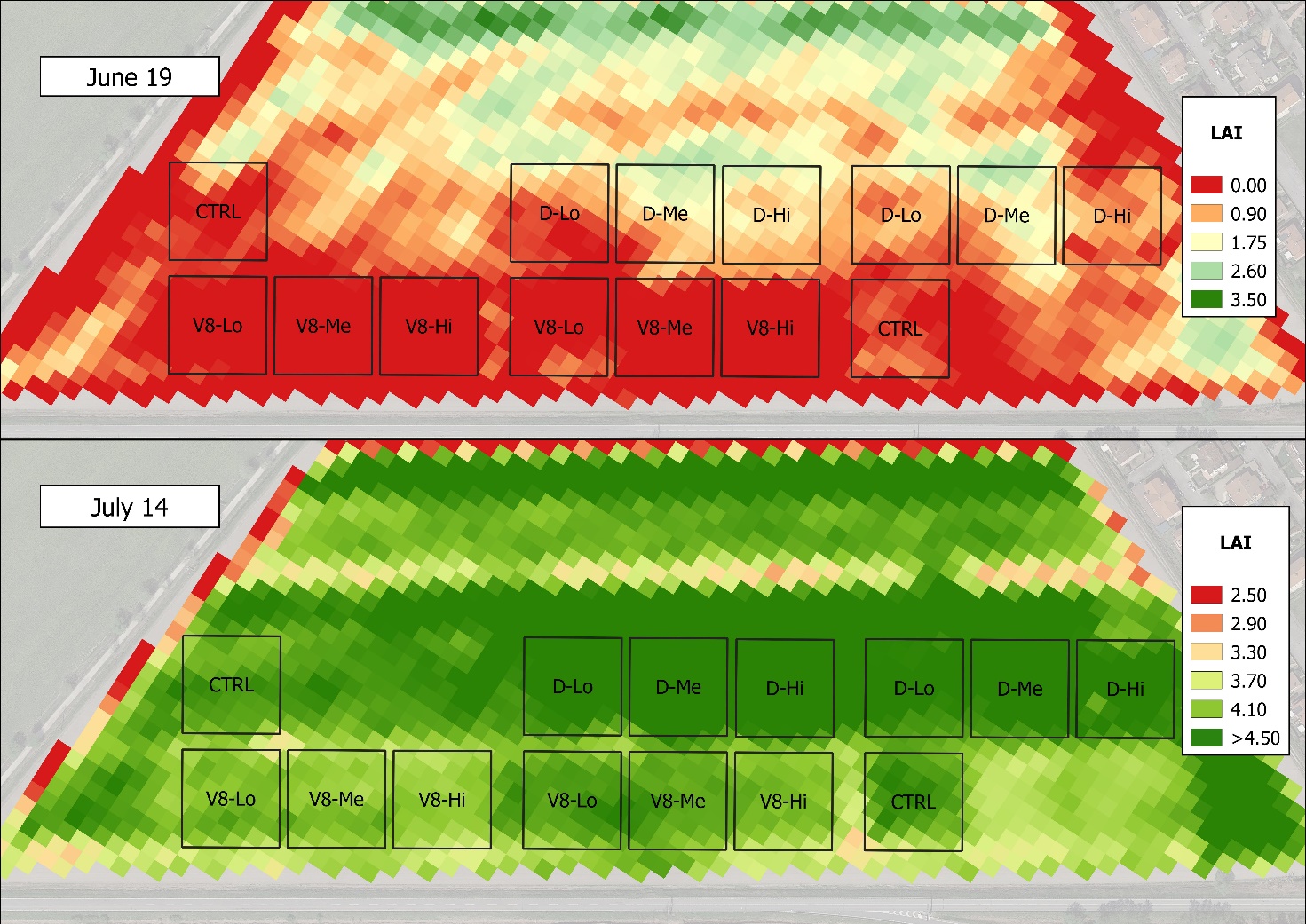


**Figure 2-SP**: Estimated LAI_S2_MLA_ map over the experimental field for the four sampling dates of June 19, July 14, August 6 and September 15. Due to different LAI ranges across the season, every date is presented with a specific legend.


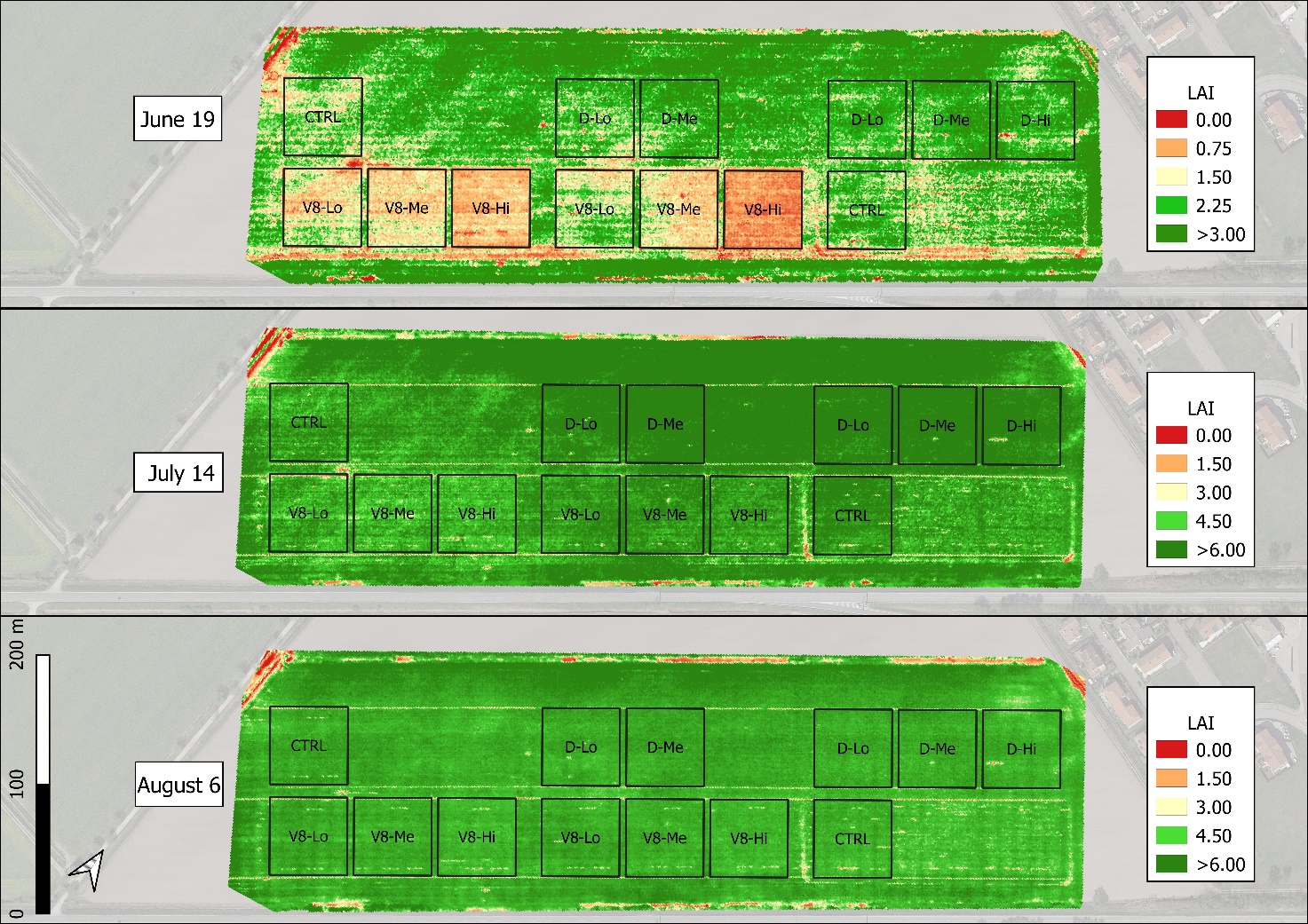


**Figure 2-SP**. Estimated LAI_UAV_ map over the experimental field for the three sampling dates of June 19, July 14 and August 6. Due to different LAI ranges across the season, every date is presented with a specific legend.
